# Influence of Water on the Oxidation of NO on Pd/TiO_2_ Photocatalysts

**DOI:** 10.3390/nano10122354

**Published:** 2020-11-27

**Authors:** M. J. Hernández Rodríguez, E. Pulido Melián, J. Araña, J. A. Navío, O. M. González Díaz, Dunia E. Santiago, J. M. Doña Rodríguez

**Affiliations:** 1 Grupo de Fotocatálisis y Espectroscopia para Aplicaciones Medioambientales (FEAM. Unidad Asociada al CSIC por el Instituto de Ciencias de Materiales de Sevilla), Departamento de Química, Instituto de Estudios Ambientales y Recursos Naturales (i-UNAT), Universidad de Las Palmas de Gran Canaria, 35017 Las Palmas, Spain; javier.arana@ulpgc.es (J.A.); oscar.gonzalez@ulpgc.es (O.M.G.D.); dsantiago@proyinves.ulpgc.es (D.E.S.); jose.dona@ulpgc.es (J.M.D.R.); 2 Centro Mixto CSIC-Universidad de Sevilla, Departamento de Química Inorgánica, Instituto de Ciencia de Materiales de Sevilla, Universidad de Sevilla, Avenida Américo Vespucio s/n, 41092 Sevilla, Spain; navio@us.es

**Keywords:** photocatalysis, NO_x_, TiO_2_, Pd, water, FTIR

## Abstract

Two series of new photocatalysts were synthesized based on modification with Pd of the commercial P25 photocatalyst (EVONIK^®^). Two techniques were employed to incorporate Pd nanoparticles on the P25 surface: photodeposition (series Pd-P) and impregnation (series Pd-I). Both series were characterized in depth using a variety of instrumental techniques: BET, DRS, XRD, XPS, TEM, FTIR and FESEM. The modified series exhibited a significant change in pore size distribution, but no differences compared to the original P25 with respect to crystalline phase ratio or particle size were observed. The Pd^0^ oxidation state was predominant in the Pd-P series, while the presence of the Pd^2+^ oxidation state was additionally observed in the Pd-I series. The photoactivity tests were performed in a continuous photoreactor with the photocatalysts deposited, by dip-coating, on borosilicate glass plates. A total of 500 ppb of NO was used as input flow at a volumetric flow rate of 1.2 L·min^−1^, and different relative humidities from 0 to 65% were tested. The results obtained show that under UV-vis or Vis radiation, the presence of Pd nanoparticles favors NO removal independently of the Pd incorporation method employed and independently of the tested relative humidity conditions. This improvement seems to be related to the different interaction of the water with the surface of the photocatalysts in the presence or absence of Pd. It was found in the catalyst without Pd that disproportionation of NO_2_ is favored through its reaction with water, with faster surface saturation. In contrast, in the catalysts with Pd, disproportionation took place through nitro-chelates and adsorbed NO_2_ formed from the photocatalytic oxidation of the NO. This different mechanism explains the greater efficiency in NO_x_ removal in the catalysts with Pd. Comparing the two series of catalysts with Pd, Pd-P and Pd-I, greater activity of the Pd-P series was observed under both UV-vis and Vis radiation. It was shown that the Pd^0^ oxidation state is responsible for this greater activity as the Pd-I series improves its activity in successive cycles due to a reduction in Pd^2+^ species during the photoactivity tests.

## 1. Introduction

The continuing deterioration of the environment is a problem that has been acquiring ever greater relevance over the last few decades. In recent years, there has been growing concern about the deterioration in air quality. The emission of contaminating gases into the atmosphere—principally as a result of anthropogenic activity—is one of the issues that has generated the greatest concern. The legally established limits in various cities around the world are being exceeded on a regular basis. Among the most dangerous gases are ozone and nitrogen oxides or NO_x_ [1]. These atmospheric pollutants can provoke numerous harmful chemical reactions, making their elimination a priority [2]. Heterogeneous photocatalysis has been shown to be an effective tool for such elimination, as reported in several published studies [3,4,5,6]. Various articles have demonstrated its efficiency in the elimination of NO_x_ [7,8]. Nonetheless, it is also clear that its application with solar radiation needs to be improved as the current commercially available catalysts do not show significant photocatalytic activity in this wavelength range (400–700 nm) [9]. One of the techniques that can be used to enhance this aspect is the incorporation of metals into/onto the semiconductor [9]. In this respect, catalyst modification can be internal or surface-based. With surface modification, the metals are incorporated onto the surface of the catalyst without altering its internal structure and without affecting the arrangement of atoms in the crystalline lattice of the catalyst [10,11]. As a result of previous studies [12], it is known that the incorporation of Pd onto TiO_2_ can enhance photocatalyst absorption in the visible range (400–700 nm). Pd is one of the most extensively used noble metals in heterogeneous catalysis and photocatalysis after Pt and Au to improve photocatalytic efficiency in pollutant removal in water [13] and air [14]. Lee et al. [15] compared the results that they obtained according to the nature of the TiO_2_ employed, the type of noble metal (Pd or Pt), the deposition method and the type of photocatalytic reaction. Some of the results were apparently contradictory, showing that the Pt-TiO_2_ and Pd-TiO_2_ systems were not always more effective than the unmodified TiO_2_. The number of publications related to the study of the photoactivity of Pd-modified photocatalysts has grown in recent years [16,17,18,19,20]. However, very few studies have been published on NO elimination and none on in-depth analyses of the effect of the presence of Pd on the elimination mechanism. Some publications have focused very specifically on the synthesis of new materials modified with Pd [21,22], but without placing any importance on their reactivity, while others [14,23,24] have tested modified commercial materials under very specific conditions—including some not very common values for these conditions of radiation, temperature, humidity and NO concentrations. We consider that this present work constitutes an extension and completion of these previous studies. Consequently, taking as a starting point the knowledge acquired from these studies, this work evaluates the incorporation of Pd on TiO_2_ by impregnation and photodeposition for NO_x_ removal at 500 ppb concentration, at a temperature of 25 °C and with and without humidity. Identification is made through infrared spectroscopy of the species that are formed in NO_x_ oxidation, and the influence of the water and palladium species on reactivity is discussed. In addition, visible (Vis) radiation was used as well as UV-vis, and photocatalyst stability was tested after long periods of radiation and reuse. This comprises one of the major goals of this study since photocatalyst reuse is one of the issues that need to be solved for the practical application of this technology.

## 2. Experimental

### 2.1. Synthesis of Photocatalysts

The modified TiO_2_ catalyst was Aeroxide TiO_2_ P25 (Evonik Degussa GmbH, Germany). The catalysts modified with Pd were obtained by photodeposition (P) and impregnation (I). The photodeposition of Pd was described previously by Maicu et al. [12]. The P25 was suspended in water at a concentration of 5 g·L^−1^. A total of 400 mL of the suspension was taken, and 9.2 mL of isopropanol added, and 0.5% in weight of Pd with respect to the TiO_2_ from palladium (II) nitrate dihydrate (Sigma-Aldrich). The mixture was placed under a low continuous flow of N_2_ to maintain an inert atmosphere, illuminated for 6 h with a medium-pressure 400 W mercury lamp (2.6 × 10^−7^ Einstein·s^−1^·L^−1^) (Photochemical Reactors Ltd.) with emission principally in the UV-A region, and kept refrigerated at a temperature below 293 K. The suspensions were filtered using a 0.45 µm filter and dried at 383 K for 24 h. Pd impregnation was described by Wu et al. [14]. In brief, 4 g of P25 were mixed with 50 mL of a Pd(NO_3_)_2_/HCl solution (40 mg in 100 mL HCl 36% *v*/*v*). Contact was maintained for 48 h under stirring, with subsequent drying at 333 K for 12 h.

### 2.2. Dip Coating

The catalysts were deposited onto previously washed 50 cm^2^ borosilicate 3.3 plates with a dip-coating procedure (KSV-DC Dip-Coater). The plates were introduced into a 14 g·L^−1^ suspension of the catalyst in methanol at a rate of 500 mm·min^−1^, left in the suspension for 20 s and withdrawn at a rate of 120 mm·min^−1^. These cycles were repeated in each case until the desired catalyst mass was reached (1.16 ± 0.01 mg·cm^−2^). The plates were dried at 373 K for 2 h.

### 2.3. Analysis Techniques

Analyses of the crystalline structure were performed by X-ray diffraction (XRD Bruker D8 Advance) with a monochromatized source of Cu Kα_1_ radiation (λ = 0.15406 nm) at 1.6 kW (40 kV, 40 mA); samples were prepared by placing a drop of a concentrated ethanol dispersion of particles onto a single-crystal silicon plate. Crystal sizes in the different phases were estimated from line broadening of the corresponding X-ray diffraction peaks using the Scherrer equation. Anatase-rutile fractions were calculated, taking into account the relative diffraction peak intensities of crystalline planes (101) and (111) of anatase and rutile, respectively. The morphology of the samples was studied by transmission electron microscopy (TEM) using a Philips CM 200 instrument operating at 200 kV and a nominal structural resolution of ~0.25 nm. BET surface area, pore volume and pore size measurements were carried out by N_2_ adsorption at 77 K using a Micromeritics 2010 instrument. Diffuse reflectance spectra were recorded on a Varian Cary 5 spectrometer equipped with an integrating sphere using PTFE (poly-tetra-fluoroethylene) as a reference to study light absorption properties of the samples [25]. Field emission scanning electron microscopy (FESEM) images were obtained using a Hitachi S-4800 microscope to determine the thickness of the deposit on the plates. Surface characterization by X-ray photoelectron (XPS) was conducted on a SPECS spectrometer, working with a constant pass energy of 40 eV. The spectrometer main chamber was maintained at a pressure of 5–6 × 10^−10^ bar, and the machine was equipped with a PHOIBOS 150 9MCD hemispherical electron analyzer, using Al Kα (hv) 1486.6 eV at 250 W and 12.5 kV. The carbon 1 s signal (284.6 eV) was used as the internal energy reference in all measurements. All photoelectron spectra were analyzed using Casa-XPS software. A Thermo Scientific-Nicolet iS10 spectrophotometer was used for the FTIR studies. The catalyst was placed between two CaF_2_ mirrors, and a 4000–1000 cm^−1^ measurement range, 2 cm^−1^ resolution and forward/backward mirror speeds of 10 kHz/6.2 kHz, respectively, were used.

### 2.4. Photoactivity Study

The photoactivity tests were performed as described in previous studies where a scheme of the setup is presented [26]. The 500 ppb concentration of NO was obtained from dilution with the air of a 100 ppm concentration of NO (supplied by Air Liquide). Humidity was added by passing the appropriate amount of dilution air through a thermostatic water bath and was controlled by means of a Rotronic HygroPalm probe. All workflows were controlled with mass flow controllers. The borosilicate glass plates were placed in a nylon reactor with a borosilicate window and a headspace of 0.5 cm. The total workflow was 1.2 L·min^−1^ at 1 atm. The light source employed was a 10 W·m^−2^ Osram Ultra Vitalux 300 W lamp. For the tests with visible light, a 420 nm Schott cutoff filter was placed between reactor and lamp. The NOx quantification was followed with a Horiba APNA-370 N/S analyzer. The results shown are the average of two tests with a maximum deviation of 5%. In all cases, the system is kept in the dark for 30 min to reach adsorption equilibrium and then the lamp is turned on (t = 0). The results are expressed as percentages of [NO] _removed_, [NO_2_] _generated_ and [NO_x_] _removed_ in accordance with the following equations:

(1) $${\mathrm{NO}}_{\mathrm{removed}}/\%=\frac{\int_{0}^{t}\left({\left[\mathrm{NO}\right]}_{o}-{\left[\mathrm{NO}\right]}_{t}\right)\mathrm{dt}}{\int_{0}^{t}{\left[\mathrm{NO}\right]}_{o}\mathrm{dt}}\cdot 100$$

(2) $${\mathrm{NO}}_{2_{\mathrm{generated}}}/\%=\frac{\int_{0}^{t}\left({\left[{\mathrm{NO}}_{2}\right]}_{t}-{\left[{\mathrm{NO}}_{2}\right]}_{o}\right)\mathrm{dt}}{\int_{0}^{t}{\left[\mathrm{NO}\right]}_{o}\mathrm{dt}}\cdot 100$$

(3) $${\mathrm{NO}}_{x_{\mathrm{removed}}}/\%=\frac{\int_{0}^{t}[\left({\left[\mathrm{NO}\right]}_{o}+{\left[{\mathrm{NO}}_{2}\right]}_{o}\right)-\left({\left[\mathrm{NO}\right]}_{t}+{\left[{\mathrm{NO}}_{2}\right]}_{t}\right)]\mathrm{dt}}{\int_{0}^{t}{\left[\mathrm{NO}\right]}_{o}\mathrm{dt}}\cdot 100$$

where [NO]_o_ and [NO_2_]_o_ are the concentrations are the concentrations at the inlet of the reactor once the adsorption equilibrium has been reached, and [NO]_t_ and [NO_2_]_t_ are the concentrations at the outlet of the photoreactor at any time under illumination.

## 3. Results

### 3.1. Characterization of the Modified Catalysts

**Figure 1:**
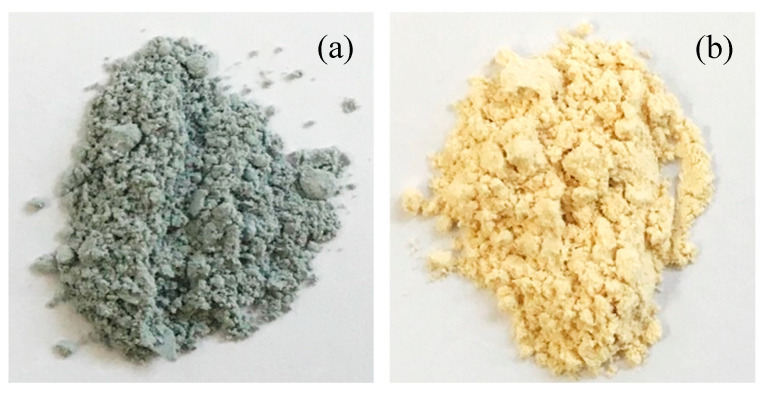
Photocatalysts Pd-P (**a**) and Pd-I (**b**).

Table 1 shows the characterization of the P25 and Pd-modified catalysts. Modification of the P25 photocatalyst through the incorporation of Pd does not result in changes in the crystalline structure of the photocatalyst. The anatase percentage is maintained, and the anatase/rutile crystallite sizes barely vary. The diffractograms can be consulted in Supporting Information. The TiO_2_ crystalline phases present in all the catalysts are anatase (A) and rutile (R), identified by the Crystallography Open Database (COD) as COD9015929 and COD9015662, respectively. No presence of metal is observed, as expected for the weight percentage of 0.5% of Pd, an amount which is difficult to detect using X-ray diffraction. Differences are observed in the colors of the Pd-modified photocatalysts (Figure 1). The Pd-P is grayish in color, whereas the Pd-I is more yellowish, indicating the presence of Pd^0^ in the Pd-P [14].

**Figure 2:**
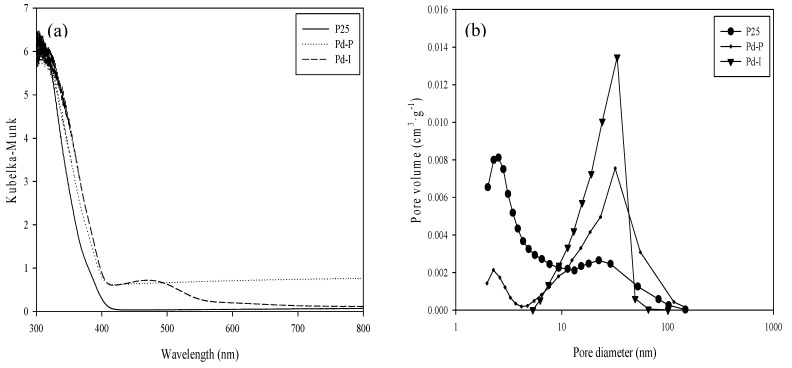
(**a**) UV-vis diffuse reflectance spectra and (**b**) Pore volume distribution of the catalysts.

Figure 2a shows the DRS UV-vis for the Pd-modified photocatalysts. Comparing the P25 spectrum with the modified samples, similar behavior is observed with a slight shift from 400 nm to longer wavelengths (“redshift”) in the sample order Pd-P > Pd-I > P25. Pd-I has a broad band between 450 and 550 nm centered at 470 nm due to a d-d transition of Pd^2+^ [27], responsible for the yellowish-brown color of the photocatalyst. Pd-P does not display bands characteristic of Pd in the visible range, although its absorbance is higher than for P25 in all the visible range due to its grayish color.

Table 1 shows the surface area values obtained after modification of the P25 with Pd. No significant changes are observed, as also reported by other authors [24]. It is seen that the incorporation of metal particles at these low percentages does not entail any significant change in the surface area of the bare TiO_2_ (P25). However, in the pore size distribution represented in Figure 2b, there can be seen an increase of the high interparticle mesoporosity in detriment to the low intraparticle mesoporosity, with this being more noticeable for Pd-I than for Pd-P. This would seem to suggest that the metal deposits have occupied the pores of lesser volume, as has also been observed for other metals such as Pt and Au in previous studies [28,29].

**Figure 3:**
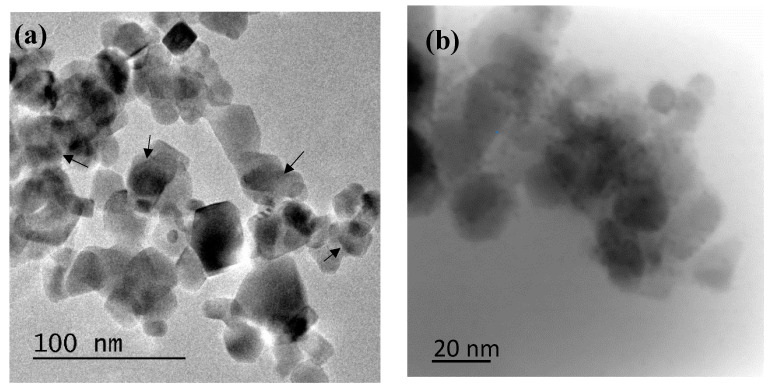
TEM of Pd-modified P25: Pd-P (**a**) and Pd-I (**b**). The latter was obtained using high-angle annular dark-field imaging (HAADF).

The TEM study enabled the identification of the deposits of Pd particles incorporated on the P25. It can be seen for both samples that the deposits are very small at the nanometer scale (Figure 3).

**Figure 4:**
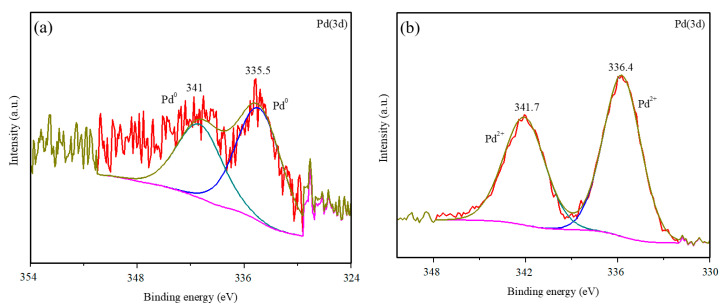
XPS of Pd-modified samples: (**a**) Pd (3d) of Pd-P and (**b**) Pd (3d) of Pd-I.

The samples were analyzed by XPS in order to determine the oxidation states of the Pd incorporated on the surface of the titanium oxide. Table 2 shows the energies of the constituent elements of the different catalysts. For all the photocatalysts, the binding energy of the Ti was 458.0 ± 0.5 eV, corresponding to the Ti^4+^ signal of the TiO_2_ (9). The O signal appears at values close to 529.5 ± 0.5 eV, attributable principally to the oxygen of the crystalline lattice of the TiO_2_ [30]. A slight shift in the Ti and O signals was detected with respect to the P25 due to the surface inclusion of the Pd. This has previously been observed with the incorporation of other metals (Pt and Au) onto P25 [31]. The O_lattice_/Ti ratio remained close to the theoretical value at around 2 in the modified photocatalysts. This indicates that the modification carried out in this work by means of the deposition of palladium particles is only superficial. An analysis of the oxidation state of the Pd can be made from Figure 4.

Figure 4a,b show the 3d spectra of the Pd for Pd-P and Pd-I, respectively. For Pd-P, the predominant species is the metallic Pd (335.5 eV (*3d*_5/2_)) [32]. Despite the small amount of metal in the samples—which makes the peak-background ratio low—a certain asymmetry can be observed due to a shoulder at higher binding energies that could be due to the presence of Pd^2+^, as described in other samples obtained by photodeposition [12,14,21]. For Pd-I, it can be seen that the doublet corresponding to the Pd^2+^ (336.4 eV (*3d*_5/2_)) is clearly defined, apparently in the form of PdO according to its value [30].

### 3.2. Characterization of the Deposits

**Figure 5:**
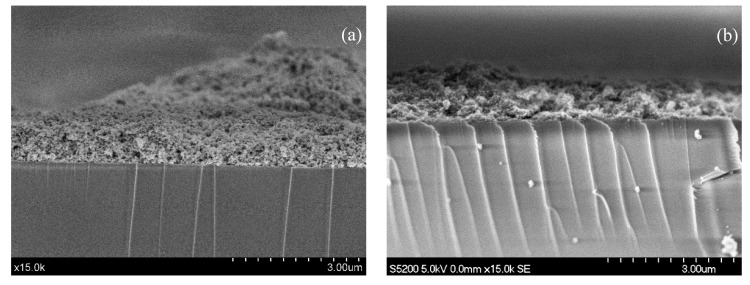
Deposit of 1.16 mg·cm^−2^ of Pd-P (**a**) and Pd-I (**b**).

The deposits of 1.16 mg·cm^−2^ of P25 with Pd, independently of the modification method, display a continuous profile irregular in thickness, ranging between 198-1690 nm and 150–1437 nm, for Pd-P and Pd-I, respectively (Figure 5). Using ASTM Standard D3330/D3330M, it was determined that the Pd deposits show good adhesion, similar to the unmodified P25 deposit.

### 3.3. Comparison of Oxidation of the NO_x_

**Figure 6:**
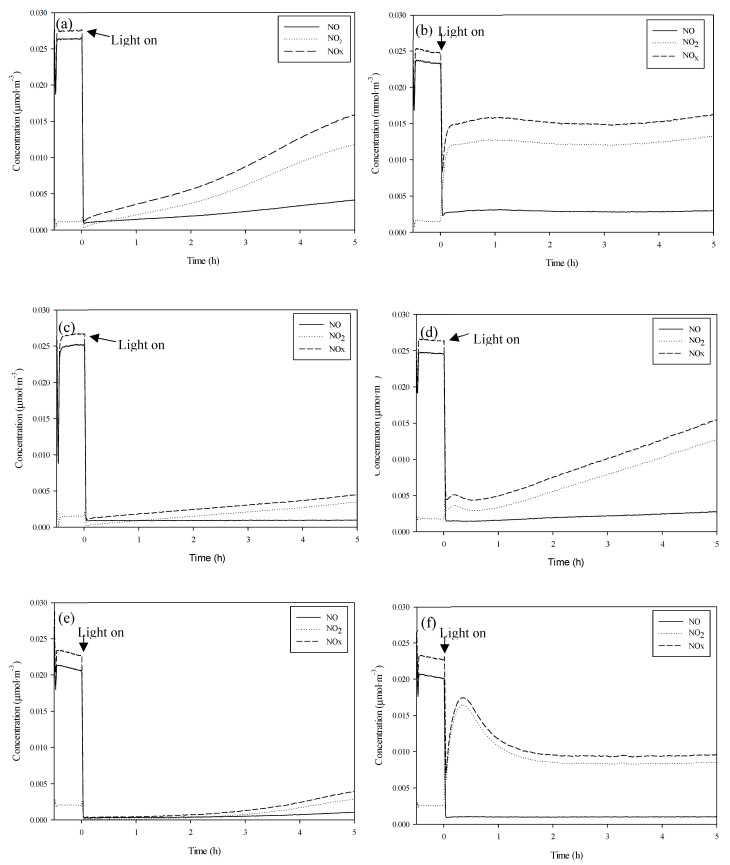
Evolution of species for P25 0% RH (**a**), P25 65% RH (**b**), Pd-P 0% RH (**c**), Pd-P 65% RH (**d**), Pd-I 0% RH (**e**) and Pd-I 65% RH (**f**).

Table 3 shows the photoactivity results obtained according to [NO] _removed_, [NO_2_] _generated_ and [NO_x_] _removed_ for Pd-P, Pd-I and P25 for comparison purposes. Reactivity was tested with different degrees of humidity. The test profiles are shown in Figure 6.

It can be observed from Table 3 that the presence of Pd entails an enhancement in NO_x_ removal both in the absence and presence of humidity. The percentage of removed NO remains practically unaltered for all the photocatalysts, independently of the relative humidity being tested. With respect to the generated NO_2_, it can be seen that, in general, as the humidity increases, more NO_2_ is generated.

**Figure 7:**
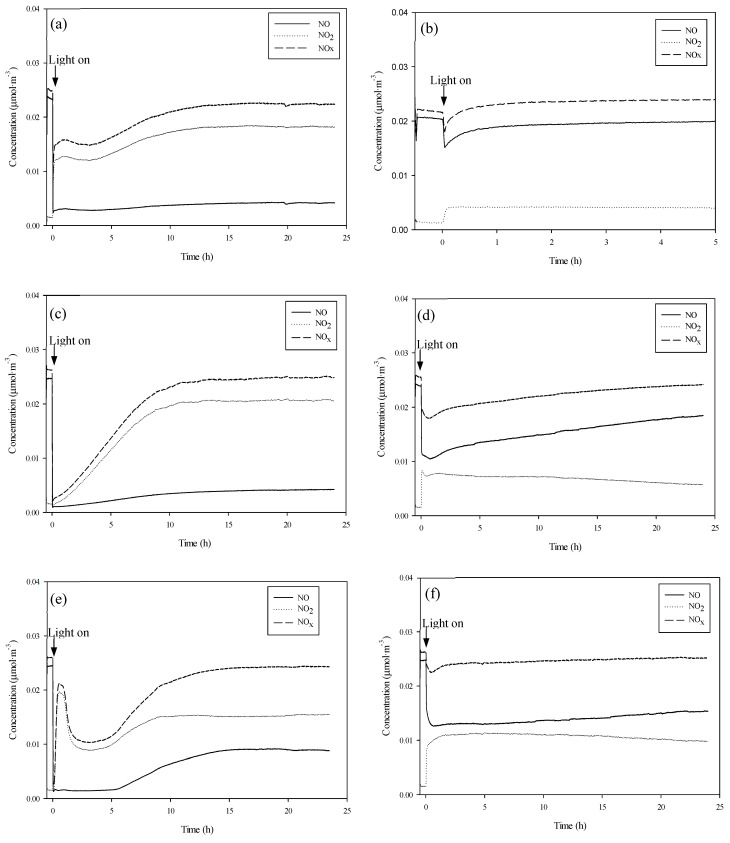
Evolution of species (65% RH): P25 UV-vis (**a**), P25 Vis (**b**), Pd-P UV-vis (**c**), Pd-P Vis (**d**), Pd-I UV-vis (**e**) and Pd-I Vis (**f**).

Longer studies of up to 24 h duration were undertaken of UV-vis irradiation under more adverse conditions of 65% RH (Figure 7a,c,e) in order to evaluate the capacity for NOx elimination of the catalysts before the occurrence of surface saturation by HNO_3_ [33]. It was found that both Pd-I and Pd-P have a superior NOx removal capacity than that of P25. The P25 had a removal rate of 26% against the 35% of Pd-I and the 37% de Pd-P.

In the following subsection, an in-depth analysis is made using infrared spectroscopy studies of the effect of the presence of Pd on the changes in the reactivity of the P25.

#### 3.3.1. Influence of the Presence of Water on the Catalyst Surface on the Oxidation Mechanism

**Figure 8:**
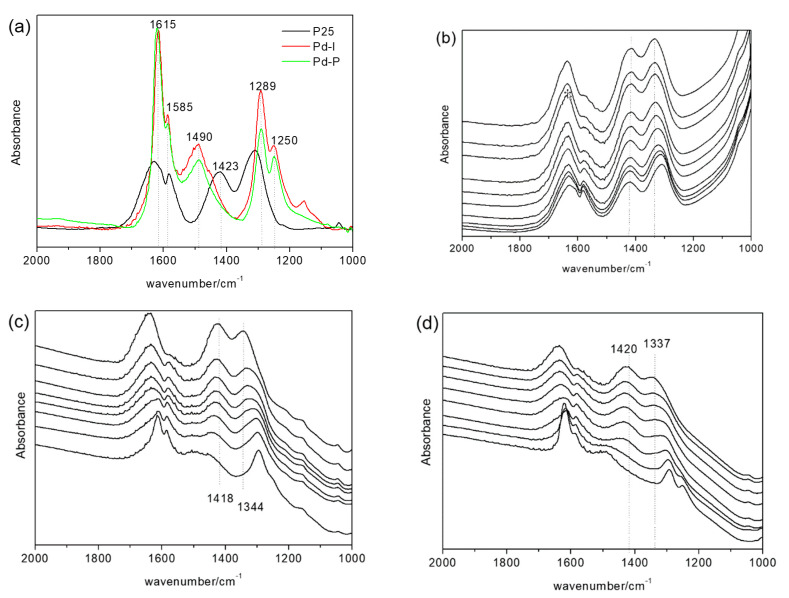
Initial FTIR spectra (**a**) and spectra obtained after 24 h of reaction at 0% RH of the P25 (**b**), Pd-P (**c**) and Pd-I (**d**).

Figure 8 shows the spectra obtained of the surface of the P25, P25-P and P25-I catalysts after the NO elimination studies. The spectra obtained with the P25-P and P25-I catalysts are practically the same, with bands centered at 1615, 1585, 1490, 1289 and 1250 cm^−1^. The P25-P spectrum additionally presents a shoulder over 1450 cm^−1^. The P25 spectrum presents a band at 1620 cm^−1^ with a shoulder at 1615 cm^−1^ and bands at 1585, 1423 and 1309 cm^−1^. These catalysts were left without illumination in an air atmosphere, and the progressive evolution of the bands observed initially was observed (Figure 8b–d). It could be seen in all cases that the initial bands progressively decreased at the same time as two new bands formed centered around 1416–1425 and 1330–1340 cm^−1^.

The bands that can be seen in the P25-P and P25-I at 1490 and 1289 cm^−1^ are attributed to nitro-chelates [34], while those observed at 1615, 1585 and 1250 cm^−1^ can be attributed to adsorbed NO_2_ [35,36]. The bands generated after concluding the test, centered around 1416–1425 and 1330–1340 cm^−1^, are attributed to solvated nitrates [37]. The analysis of these spectra suggests that, in the P25, after the NO elimination studies, the surface is saturated with solvated nitrates and, to a lesser extent, with nitro-chelates and adsorbed NO_2_. In contrast, in the catalysts with Pd, the surface is only saturated with nitro-chelates and adsorbed NO_2_.

The reaction of the NO with oxygen surface ions gives rise to nitrites (4), and the reaction of nitrites with oxygen surface ions gives rise to NO_2_ (5). Therefore, the nitrocompounds and the adsorbed NO_2_ observed in the FTIR studies (Figure 8) are generated from the reactions. Ruggeri et al. [38] described how the reaction between nitrites and NO_2_ to give nitrates, and NO constitutes an equilibrium (6). Hence, during the NO elimination studies in the catalysts with Pd, the reaction expressed in (6) does not take place until the catalysts are left in an air atmosphere, and the NO concentration decreases. However, in the case of the P25, the formation of nitrates is favored through another mechanism in which surface hydration of the catalyst is influential. This will be discussed below.

(4) Obr  .−(ads)+NO(ads)→  NO2(ads)−+     ⏟Oxygen Vacancy

(5) $$NO_{2\left(ads\right)}^{-}+O_{br\left(ads\right)}^{\;.\;-}\overset{\;}{\to }NO_{2\left(ads\right)}+O_{br}^{=}$$

(6) $$NO_{2\left(ads\right)}+NO_{2\left(ads\right)}^{-}\overset{\;}{⇄}NO_{3\left(ads\right)}^{-}+NO_{\left(ads\right)}$$

**Figure 9:**
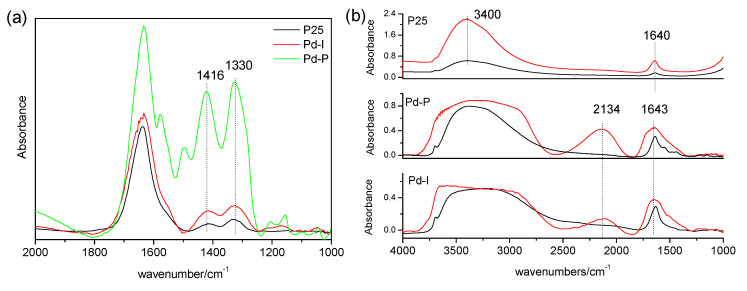
(**a**) FTIR spectra obtained after 15 h of the reaction of NO elimination at 65% RH. (**b**) FTIR spectra of the catalysts (black) and the catalysts subjected to a pure airflow at 65% RH (red).

The FTIR spectra of the catalysts after 15 h of NO elimination at 65% RH are shown in Figure 9a). Bands are mainly observed centered at 1416 and 1330 cm^−1^ attributed to solvated nitrates. These bands were more intense in the catalysts with Pd.

In the presence of humidity, the NO_2_ can be disproportionated through the following reaction:

(7) $$2NO_{2\left(ads\right)}+H_{2}O_{\left(ads\right)}\overset{\;}{\to }HONO_{\left(ads\right)}+HNO_{3\left(ads\right)}$$

It has been reported that the disproportionation of NO_2_ with water requires the formation of a prior isomer [39]. The formation of this isomer and the subsequent disproportionation of NO_2_ can be conditioned by the characteristics of the adsorbed water. Figure 9b shows the spectra of the catalysts and their spectra after subjecting them to a pure airflow at 65% RH. The P25 spectrum is represented by an intense band at 3400 cm^−1^ and another at 1620 cm^−1^ attributed to the stretching and bending modes of adsorbed water, respectively [40]. In contrast, in the catalysts with Pd, the spectrum is dominated by a broad band between 3600 and 2900 cm^−1^, another at 2134 cm^−1^ and a third at 1643 cm^−1^, which are typical of liquid water. That is, the adsorption of water in these catalysts seems to be less intense than in the P25. The intense interaction of water in the P25 appears to favor the disproportionation of NO_2_ through reaction (4).

These results are in agreement with those of previous studies made by our research team [41] in which it was found that the NO_2_ is not photocatalytically degraded but rather adsorbed and disproportionated on the surface of the TiO_2_.

In short, the absence of high surface hydration favors the formation of nitro-chelates and the adsorption of NO_2_ in the catalysts with Pd. This explains the greater eliminating capacity of this compound with the Pd-modified catalysts.

#### 3.3.2. Influence of Palladium Oxidation State

The differences with respect to the Pd-I and Pd-P series of photocatalysts come from the amount of generated NO_2_. The increase of NO_2_ generated with humidity is greater for the Pd-I series. This means that, in 5 h, NO_x_ removal is lower for Pd-I. Hence, in the absence of humidity, Pd-I and Pd-P function practically the same, but in the presence of humidity, the Pd-P series has greater photocatalytic efficiency. Although it was found for 65% RH that they have the same overall NOx removal capacity, the oxidation kinetics are faster for the Pd-P series (Figure 7c,e). Even though palladium deposition by photodeposition was less efficient (Table 2), the activity results of the P25-P series are better.

According to Sheng et al. [24], during irradiation, the formation of Pd^4+^ species takes place (8 and 9). The formation of these species is favored by the presence of humidity from the oxidation of Pd^2+^. The formation of these species and the consequences for the adsorption of NO_x_ species may explain the changes in growth and decay in the NO_2_ profile that takes place in the first hour of irradiation for the catalysts of the Pd-I series, which are the catalysts which have a higher amount of oxidized palladium (Figure 6f).

(8) $$Pd^{2+}+2HO_{ads}^{\cdot }\to Pd^{4+}+2OH^{-}$$

(9) $$3Pd^{2+}+2O_{2,ads}^{.-}+4H_{2}O\to 3Pd^{4+}+8OH^{-}$$

**Figure 10:**
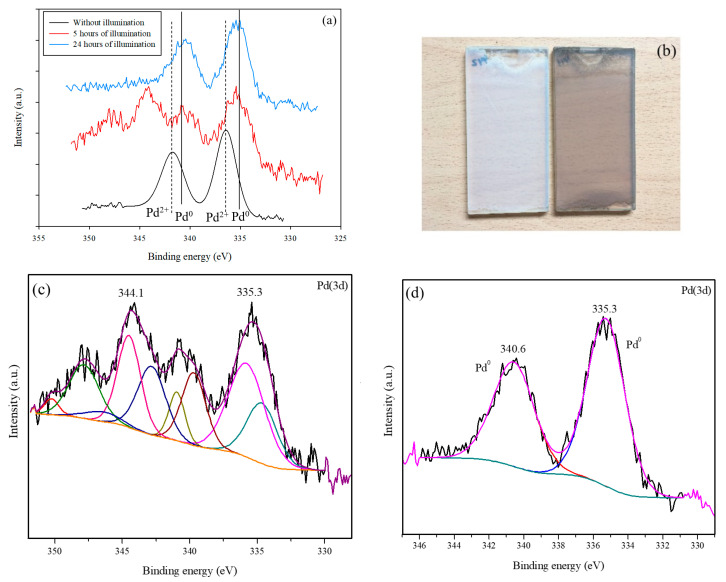
XPS spectra corresponding to the 3d region of the Pd-I: comparison at different exposure times (**a**) and deconvolution at 5 h (**c**) and 24 h of illumination (**d**). Appearance of the coatings of initial Pd-I and Pd-I after 24 h (**b**).

In order to clarify the results and the different evolution in the NO_x_ oxidation kinetics in Pd-I and Pd-P, an XPS analysis was undertaken of the evolution of the Pd-I sample for different test times of 5 h and 24 h (Figure 10a).

No appreciable differences were observed in the Ti and O signals compared to the initial sample. Figure 10c,d shows the deconvoluted Pd signals at 5 h and 24 h. A shift can be observed of the doublets corresponding to the Pd with the test time to lower binding energies (from 336.4 to 335.5 eV and from 341.7 to 340.6 eV). This corresponds to the reduction from Pd^2+^ to Pd^0^. Figure 10b shows the initial deposit of Pd-I and after 24 h of radiation. A variation in color can be observed due to the majority appearance of Pd^0^ (grayish color). For Pd-I at 5 h, Pd presents a quite complex set of peaks, which indicates more species than Pd^0^ and PdO, which may be due to the Pd^4+^ (R5 and R6).

Table 4 shows removed NO, generated NO_2_ and removed NO_x_ for various cycles of photoactivity with intermediate washes for Pd-I. Enhancement of the activity after each radiation cycle is confirmed as the Pd is reduced. In the second cycle, similar values to those of the Pd-P sample are obtained, and in the third cycle, the results are even bettered as the Pd-P sample also initially contained some oxidized Pd.

Activity was also tested with Vis radiation (Figure 7b,d,f). It can be seen that the incorporation of the metal improves P25 efficiency, especially for Pd-P with which more NO_x_ is removed and less NO_2_ generated. Activity in the visible is attributable to the presence of Pd^2+^ and Pd^0^ on the surface of the modified photocatalysts. Under visible radiation, the presence of Pd^0^ gives rise to energy transitions between the TiO_2_ valence band and localized energy levels due to metal clusters [42,43]. In addition, the PdO band gap, presumably formed in Pd-I, is low, at 0.8 eV [44], which could act as a Vis-photosensitizer [45].

## 4. Conclusions

The presence of Pd on the TiO_2_ surface improves NO_x_ removal. This improvement appears to be related to the presence of Pd, giving rise to a different interaction of the water with the TiO_2_ surface.

It is shown, as also indicated in previous studies, that NO_2_ is not photocatalytically degraded but rather is adsorbed and disproportionated on the TiO_2_ surface. In the present work, it was found that in the catalysts with Pd, the NO_2_^-^ and NO_2_ generated from the photocatalytic oxidation of the NO in the absence of humidity are adsorbed on the surface of the catalyst, and there is no disproportionation to give nitrates until the NO is eliminated from the reaction medium. However, in the P25, there is some NO_2_ disproportionation due to the high surface hydration of the catalyst and less NO_2_ adsorption.

The photodeposition method produces a higher quantity of Pd^0^ than impregnation. It seems to be that the Pd^0^ form is beneficial for the activity of the catalyst under both ultraviolet and visible radiation.

## Figures and Tables

**Table 1:** Main characteristics of the catalysts.

Catalysts	% Anatase	Crystallite Size		Surface Area (m^2^∙g^−1^)	Pore Volume (cm^3^·g^−1^)
		Anatase (nm)	Rutile (nm)		
P25	82.0	23.0	33.0	48.6	0.18
Pd-P	79.4	21.2	35.4	49.8	0.41
Pd-I	81.7	18.9	33.1	52.7	0.33

**Table 2:** XPS results of the Ti, O and Pd regions for the different modified photocatalysts.

Catalysts	Binding Energy/eV (Ti)	% at. Ti	Binding Energy/eV (O)	% at. O	Binding Energy/eV Pd	% at. Pd	% wt. Pd	O_lattice_/Ti
Pd-P	458.01	28.35	529.01	71.53	334.01	0.11	0.37	2.10
Pd-I	458.00	28.38	529.00	71.32	336.00	0.27	1.05	2.20

**Table 3:** Percentages of NO removed, NO_2_ generated, and NO_x_ removed for each of the tested photocatalyst samples for 5 h of UV-vis irradiation. RH stands for relative humidity.

	NO Removed (%)	NO_2_ Generated (%)	NO_x_ Removed (%)		NO Removed (%)	NO_2_ Generated (%)	NO_x_ Removed (%)
Pd-P 0% RH	96.23	1.17	95.06	Pd-I 0% RH	97.05	0.00	97.05
Pd-P 25% RH	95.04	3.61	91.43	Pd-I 25% RH	95.05	17.77	77.28
Pd-P 40% RH	89.69	20.74	68.95	Pd-I 40% RH	93.90	38.40	55.50
Pd-P 65% RH	91.27	21.19	70.08	Pd-I 65% RH	95.01	34.54	60.47
P25 0% RH	90.73	16.29	74.44	P25 65% RH	87.16	46.50	40.65

**Table 4:** Percentages of NO removed, NO_2_ generated, and NO_x_ removed for 4 reuse cycles of Pd-I and 5 h of UV-vis irradiation.

	NO Removed (%)	NO_2_ Generated (%)	NO_x_ Removed (%)
**Cycle 1**	95.01	34.54	60.47
**Cycle 2**	92.18	20.53	71.65
**Cycle 3**	92.33	9.18	83.14
**Cycle 4**	94.16	8.11	86.05
